# The effect of maternal position on cerebral oxygenation in premature infants during Kangaroo care: a randomised controlled trial

**DOI:** 10.1038/s41372-025-02287-0

**Published:** 2025-04-05

**Authors:** Iyshwarya Stapleton, Sarah Murphy, Susan Vaughan, Brian Henry Walsh, Kannan Natchimuthu, Vicki Livingstone, Eugene Dempsey

**Affiliations:** 1 Department of Neonatology, Cork University Maternity Hospital, Cork, Ireland; 2 Irish Centre for Maternal and Child Health Research (INFANT) Centre, University College Cork, Cork, Ireland; 3 Department of Paediatrics and Child Health, University College Cork, Cork, Ireland

**Keywords:** Paediatrics, Preventive medicine

## Abstract

**Objective:**

To assess whether there was an optimal maternal position (30° versus 60° incline) for kangaroo mother care.

**Design:**

Single centre cross-over randomised controlled trial. Mothers were randomly assigned to start at either a 30° or 60° angle. Primary outcomes were the mean cerebral near-infrared spectroscopy (NIRS) values. Secondary outcomes included median peripheral saturations and heart rates.

**Results:**

Twenty infants were included in the final analysis: median gestational age at birth was 28^+1^ weeks and median birth weight was 985 g. No significant differences were observed in the primary outcomes or the secondary outcomes at either angle.

**Conclusions:**

Maternal positioning at a 30° or 60° incline did not impact on cerebral oxygenation values in very preterm infants. Either position was associated with clinical stability.

****Trial Registration Number**:**

ClinicalTrials.gov ID NCT05686252.

## Introduction

Kangaroo Mother Care (KMC) involves establishing skin-to-skin contact between infant and parent and has been demonstrated to be of significant benefit. For low-birth weight infants, it has been demonstrated to aid physiological stability. In addition, KMC has been demonstrated to reduce the incidence of mortality, severe illness, infection, and length of hospital stay [1–3]. KMC has been shown consistently to aid establishment of breastfeeding and thus plays a substantial role in aiding nutrition and development [4, 5]. A recent Cochrane review found that KMC reduced mortality at discharge [1]. KMC increased weight, length, and head circumference, and breastfeeding rates at discharge and at one to three months’ follow-up [1]. An earlier study found that morbidity, mortality, growth, development, and other selected health-related outcomes were at least as good as or better than usual care when infants reached term and at 1-year corrected age (CA) [6]. A 20 year follow up study of this large, randomised control trial found that the positive effects of KMC at 1 year on IQ and home environment were maintained [7]. Notably neuroimaging demonstrated increased volume of the left caudate nucleus in the KMC group [7]. These findings have been recently confirmed in another study which compared new-born infants that received early KMC (defined as at <72 h of life) and prolonged KMC to neonates without early or prolonged KMC [3]. Those who received early initiation and prolonged KMC had better neurodevelopmental outcomes including language, cognition and adaptive behaviour at a corrected age of 12 months [3].

A positive correlation between duration in the kangaroo position (the longer in the position, the larger the volume of the caudate nucleus) and the results of the fine motor skills test (the better the performance, the larger the volume of the caudate nucleus) at 20 years of age have been identified [7].

A recent large international randomised control trial has demonstrated that those infants who received immediate kangaroo mother care had lower mortality at 28 days than those who received conventional care with kangaroo mother care initiated after stabilization [8]. Other studies found that KMC reduced maternal anxiety and depression in infancy, improved attention in infancy and executive functions in children, along with increased mother–child harmony [9]. In addition to recommending KMC as routine care for all preterm or low-birthweight infants, the World Health Organisation (WHO) also recommends commencing KMC for pre-term or low-birth-weight infants as soon as possible after birth [10].

It is generally accepted that if a baby is held whilst in an elevated tilt position it may reduce the risk of hypoxaemic/bradycardic episodes [11]. Little data exists on the optimal held position during KMC and its impact on cerebral oxygenation. Thus, we designed a randomised control trial comparing being held at 30° or 60° angles for KMC and its impact on cerebral oxygenation and other physiological parameters. We hypothesised that being held at 30° during KMC is superior to being held at 60° in babies born extremely premature.

## Methods

### Trial design and participants

This was a superiority cross-over randomised controlled trial performed in the neonatal intensive care unit (NICU) of a tertiary neonatal unit. It included premature infants who were a minimum of 28 weeks corrected gestational age (cGA), at least 600 g at the time of enrolment and who would have been receiving KMC as part of their routine care. We enrolled infants over a five-month period between May and September 2022.

### Ethics approval and consent to participate

This study was approved by the Clinical Research Ethics Committee of the Cork Teaching Hospitals, Cork, Ireland. Informed consent was obtained from all the parents prior to randomisation and participation in the trial. Infants with any known neurological abnormalities (other than intraventricular haemorrhage), orthopaedic conditions and/or chromosomal abnormalities were excluded.

### Sample size

An a priori sample size calculation indicated that a sample of 20 patients was necessary to detect a difference of 2% in NIRS percentage in a cross-over study using a paired t-test comparing 30° and 60° angle positions. This sample size was based on assuming a correlation of 0.5 between paired measurements, a standard deviation of difference of 3, a power of 80%, a level of significance of 0.05 and a 2-tailed test. The sample size calculation was performed using the G-Power 3.1 programme [12].

### Randomisation and procedures

**Fig. 1:**
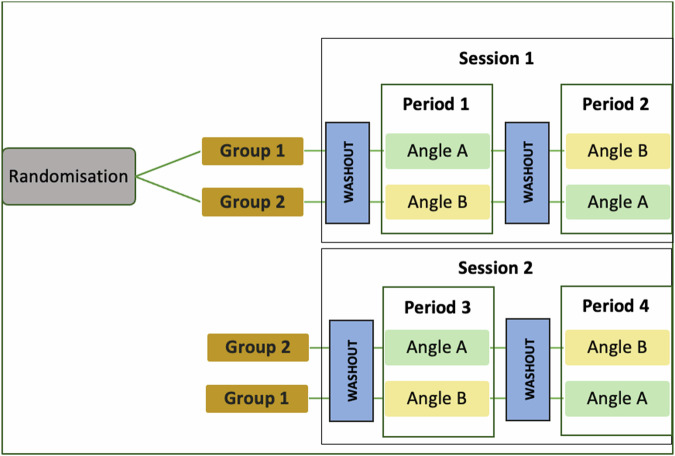
Randomisation process, timing of sessions and wash-out periods, and measurement periods.

**Fig. 2:**
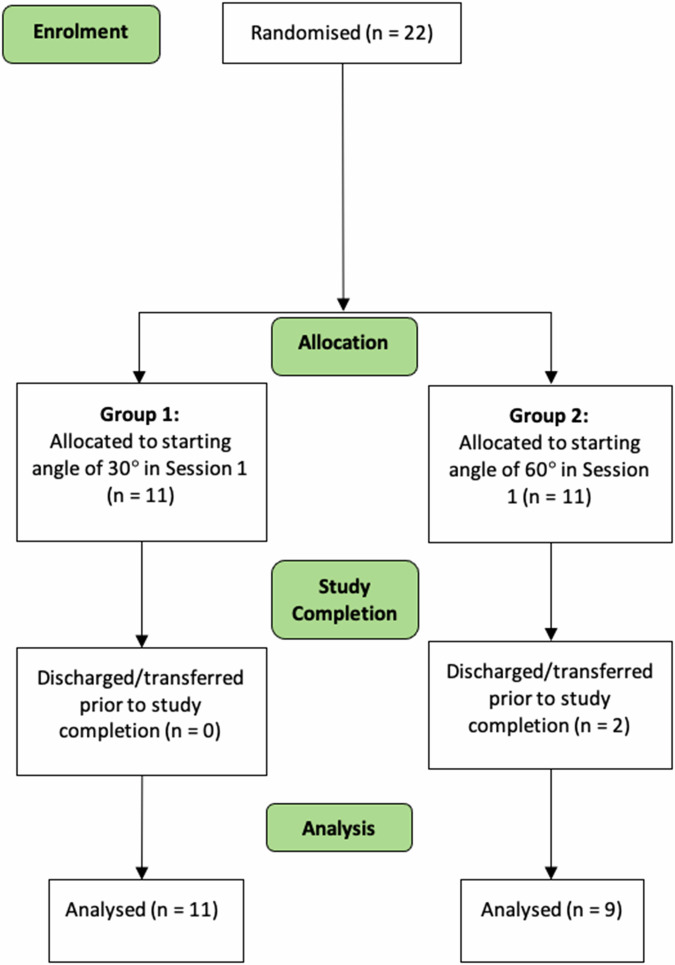
Consolidated Standards of Reporting Trials (CONSORT) flow diagram of enrolment, allocation to Group 1 or 2 and numbers analysed for the trial.

Eligible infants were enrolled by study investigators (IS, SM and SV) and randomly assigned (1:1) to one of two groups. In Group 1, infants began Session 1 at the 30° angle, followed by 60°, and Session 2 at 60°, followed by 30°. In Group 2, infants began Session 1 at the 60° angle, followed by 30°, and Session 2 at 30°, followed by 60° (see Fig. 1). Numbers were generated using a sequence generator, printed out, and stored in sealed, opaque envelopes. When an infant was enrolled, an envelope was selected. If the number inside the envelope was odd, the infant was assigned to Group 1; if the number was even, the infant was assigned to Group 2, as illustrated in Fig. 2. For each session, during routine kangaroo mother care, the NIRS probe was applied to the baby’s forehead and the baby was subsequently placed prone on the parent’s chest. The chair was then put at the starting angle to which they had been randomised and the cerebral NIRS values, heart rate and peripheral saturations were recorded for 30 minutes. The chair was then gently repositioned into the other angle and the baby was observed and the values recorded for a further 30 minutes.

### Equipment

The chairs used in this study were the Tucson Reclining Mauro Relax Ergo-line medical chair (Haelvoet NV, Ingelmunster, Belgium). These are routinely used in the NICU for the delivery of kangaroo care. This chair can be inclined to 120° at full incline which coincides with the 30° angle used in this study (information on the angles can be found at: https://www.haelvoet.com/en/products/details/354 and illustrated in Supplementary Fig. 1). The 60° angle of inclination was determined using a protractor during the design phase of this study.

Cerebral NIRS was monitored using the Masimo Root with O3 regional Oximetry machine (Masimo, Irvine, California, USA) with the Medtronic INVOS Cerebral Oximetry Infant-Neonatal Sensor (Medtronic Limited, Watford, United Kingdom).

### Outcomes

The primary outcomes were cerebral NIRS values during the KMC sessions at both 30° and 60° angles. This consisted of the mean cerebral tissue oxygenation saturation (rSO_2_) and cerebral fractional tissue oxygen extraction (FTOE) values. The rSO_2_ is measured as a percentage. The FTOE was calculated using the following formula: (SpO_2_ − rSO_2_)/SpO_2_ [13].

There were four secondary outcomes assessed during the sessions at the two angles: (1) peripheral oxygen saturation (%), (2) heart rate (beats per minute), (3) number of bradycardias <100 beats per minute, and (4) number of desaturations less than 80% lasting longer than 20 seconds.

### Statistical analysis

Categorical data was described using frequencies and percentages and continuous data using means and standard deviations (SD) (when the data was normally distributed) or as medians and inter-quartile ranges (IQR) otherwise. The effect of the angle (30° or 60°) on the continuous primary and secondary outcomes was investigated using linear mixed effects models with angle as a fixed effect and subject as a random effect. As SpO_2_ was negatively skewed, bootstrapped 95% confidence intervals (with 10 000 repetitions) were also calculated to assess the sensitivity of the results to non-normality. The conclusions did not change. The effect of the angle on the secondary binary outcomes was investigated using logistic mixed effects models with angle as a fixed effect and subject as a random effect. In the initial models, period and sequence were included as fixed effects to test for period and sequence effects. As there were no significant period or sequence effects, they were removed as fixed effects in the final model. Due to multicollinearity, carry-over effects were not investigated. The four periods were also looked at separately and outcomes compared between the two angles using the Wilcoxon signed rank test. Statistical analysis was performed using IBM SPSS Statistics (version 29.0, IBM Corp., Armonk, NY).

## Results

Of the babies that met our inclusion criteria, twenty-two were approached and gave informed consent to take part in the study. These were randomised to either group 1 or 2. Of these, two were discharged prior to completion of the second session (Fig. 1) and so were excluded from the final analysis. The remainder of the babies had both sessions completed and the data collected was analysed.

Demographic data for the twenty infants included in the trial is included in Table 1. All infants were 7 post-natal days or more at the time of their first session. The median gestational age at birth was 28^+1^ weeks, with the earliest GA at birth being 23^+2^. The median birth weight of the infants was 985 g, with the smallest baby being 620 g at birth. The baseline level of respiratory support required by the infants at the time of each of the sessions was also recorded and is outlined in Table 1.

**Table 1:** Demographic results, feeding and respiratory support for the twenty participants.

	Birth	Session 1		Session 2
Corrected gestational age: Median (IQR)	28^+1^ (26^+6^–30^+0^)	32^+6^ (31^+0^–34^+3^)		33^+2^ (31^+2^–36^+3^)
Birth Weight: Median (IQR)	0.985 kg (0.755–1.345 kg)			
**Feeding**	**Session 1**		**Session 2**	
*Median volumes (IQR)*	160 ml/kg/day (152.5–160)		160 ml/kg/day (160–160)	
*Fortified MEBM*^a^	14/20		14/20	
**Level of Respiratory Support**	**Session 1**		**Session 2**	
*Self-ventilating (%)*	8 (40%)		10 (50%)	
*CPAP*^b^ *(%)*	5 (25%)		3 (15%)	
*BIPAP*^c^ *(%)*	1 (5%)		1 (5%)	
*High-flow oxygen therapy (%)*	3 (15%)		4 (20%)	
*Low-flow oxygen therapy (%)*	3 (15%)		2 (10%)	

^a^Maternal expressed breast milk.

^b^Continuous positive airway pressure.

^c^Biphasic positive airway pressure.

### Primary outcomes

Overall, no significant differences were observed between the angles for mean cerebral rSO2 [mean (standard deviation, SD): at 30° = 65.94 (10.96); at 60° = 67.63 (8.78); *p* = 0.332] and mean cerebral FTOE values [mean (SD): at 30° = 0.30 (0.11); at 60° = 0.28 (0.09); *p* = 0.259] (Table 2). Similarly, when examining the four periods separately, no significant differences were identified between the medians at each of the angles (Table 3).

**Table 2:** Overall comparison between angles, *n* = 20.

	Observed		From linear mixed model^a^	
	30° Mean (SD)	60° Mean (SD)	Difference in means^b^ (95% CI)	*p* value
**Primary outcomes**^c^				
*rSO*_*2*_	65.94 (10.96)	67.63 (8.78)	1.69 (–1.76 to 5.14)	0.332
*FTOE*	0.30 (0.11)	0.28 (0.09)	–0.02 (–0.06 to 0.02)	0.259
**Secondary outcomes**^c^				
*SpO*_*2*_	94.78 (3.83)	94.40 (3.61)	–0.38 (–1.50 to 0.75)	0.508
*HR*	152.70 (9.87)	155.15 (8.97)	2.45 (–0.50 to 5.40)	0.102

^a^with angle as a fixed effect and subject as a random effect.

^b^60° - ‘30°.

^c^for each outcome, each subject had two measurements for each of the angles.

**Table 3:** Summary of results for primary and secondary outcomes.

Primary outcome: cerebral NIRS		30° Median (IQR)	60° Median (IQR)	*p* value
Period 1	**rSO**_**2**_ **(%)**	67 (54.5–75)	68 (59.5–73)	0.882
	**FTOE**	0.3 (0.19–0.39)	0.28 (0.23–0.36)	0.882
Period 2	**rSO**_**2**_ **(%)**	62 (58.5–71.5)	69 (63–78)	0.331
	**FTOE**	0.33 (0.25–0.37)	0.23 (0.17–0.36)	0.37
Period 3	**rSO**_**2**_ **(%)**	63 (54.5–71)	68 (57–75)	0.412
	**FTOE**	0.3 (0.24–0.39)	0.29 (0.22–0.39)	0.603
Period 4	**rSO**_**2**_ **(%)**	70 (63–75)	66 (59–72.5)	0.603
	**FTOE**	0.28 (0.23–0.34)	0.28 (0.23–0.33)	1

Secondary outcomes: Peripheral oxygen saturations (SpO_2_) and Heartrate (HR)		30° Median (IQR)	60° Median (IQR)	*p* value
Period 1	**SpO**_**2**_ **(%)**	95 (94–99)	94 (92–95.5)	0.295
	HR (bpm^*^)	157 (141–164)	154 (149–163)	0.941
Period 2	**SpO**_**2**_ **(%)**	94 (92–97.5)	95 (92–98)	0.603
	**HR (bpm)**	154 (147–160)	161 (148–163)	0.37
Period 3	**SpO**_**2**_ **(%)**	94 (90–97.5)	95 (92–98)	0.766
	**HR (bpm)**	153 (146–156.5)	155 (149–160)	0.503
Period 4	**SpO**_**2**_ **(%)**	96 (94–98)	95 (90.5–97)	0.37
	**HR (bpm)**	154 (146–157)	153 (144.5–161.5)	0.941

Secondary outcomes: Numbers of babies with Bradycardia or Desaturations		30°	60°	Both angles
Significant Bradycardic episodes (*n* = 10)		6	2	2
Significant Desaturation episodes (*n* = 6)		3	1	2

^*^Beats per minute (bpm).

### Secondary outcomes

There were no significant differences in oxygen saturations [mean (SD): at 30° = 94.78 (3.83); at 60° = 94.40 (3.61); *p* = 0.508] and heart rate [mean (SD): at 30° = 152.7 (9.87); at 60° = 155.15 (8.97); *p* = 0.102] between the two angles either overall (Table 2) or in the medians when examining the four periods separately (Table 3).

There were 17 bradycardic episodes in total in 10 different neonates. Of these, 13 of the episodes were at the 30° angle and four were at the 60° angle. The odds of a baby having a bradycardic episode did not differ by angle [OR (95% CI): 0.38 (0.11–1.37), *p* = 0.14 where 30° angle was the reference]. Two of the 10 babies that had bradycardias had an episode at each of the two angles. Of the remaining eight babies, two had a bradycardic episode at the 60° angle and six had a bradycardic episode at the 30° angle.

There were 16 desaturation episodes in total in 6 different neonates. Of these, 10 of the episodes were at the 30° angle and six were at the 60° angle. The odds of a baby having a desaturation episode did not differ by angle [OR (95% CI): 0.76 (0.18 to 3.32), *p* = 0.72 where 30° angle was the reference]. Two of the six babies had an episode at each of the two angles. Of the remaining 4 babies, three had a desaturation episode at 30° angle and one had an episode 60° angle.

## Discussion

Numerous studies have confirmed the many advantages of KMC. However, data regarding the best position in which to hold an infant during KMC is currently lacking. At present current guidelines provided by WHO do not specify a specific angle at which an infant should be positioned [10]. We found no statistically significant differences in the primary outcomes of NIRS-derived mean values for cerebral tissue oxygenation saturation (rSO_2_) and fractional tissue oxygen extraction (FTOE) whether infants were held at a 30° angle compared to a 60° angle, thus suggesting either angle can effectively be used for KMC. Reassuringly we found no statistically significant differences between the two angles for any of the other outcomes at any of the periods (*p* > 0.05 for all). In both angles, oxygen saturations were maintained within an acceptable range. There was no significant difference in number of bradycardias or desaturations. Thus, it can be deduced that KMC in two positions (60° angle which is more commonly employed) or the more prone position of 30° (as trialled in our study) are safely practicable in the NICU, although this was not the primary outcome measure that the study was powered to assess. A recent systematic review and meta-analysis has found that there was no reliable evidence that position of a preterm infant either supine or prone influences rSO_2_ in the first 2 weeks of life [14]. A subgroup analysis of this also indicated that in the second week after birth, the prone position may result in higher cerebral rSO_2_ than the supine position with head in midline [14]. Both angles were demonstrated to be safe in terms of Sp0_2_ and heart rate recordings during sessions. There were no significant increase in the number of bradycardias or desaturation events during KMC, which is in keeping with findings in a previous study, which showed a reduction in the number of apnoeic episodes during KMC [15].

Our study included 20 infants on a range of ventilatory support. For infants in their first session of the KMC study, eight were self-ventilating in room air, five on CPAP, three receiving high-flow, three on low-flow oxygen therapy and one on BiPAP. By their second KMC session, ten were self-ventilating in room air, four on high flow oxygen therapy, three on CPAP, two on low-flow and one on BiPAP. Our study did not include any infants on invasive mechanical ventilation, which was likely due to the inclusion criteria of needing to be 28 weeks’ corrected gestational age. Kangaroo care has previously been shown to be a safe and viable option for very preterm neonates while intubated, although the risk of endotracheal tube dislodgement during the movement is a factor [16].

There were several limitations of this trial. One limitation was that the observed standard deviation of the difference in the primary outcome between the two angles was much higher than what we had assumed in the sample size calculation. As a result, the study was underpowered, and the confidence interval for the difference in the primary outcome between the two angles was wide and inconclusive. In addition, a larger sample size would also be required to evaluate the secondary outcomes in order to fully establish whether one position is superior to the other. A larger sample size could also provide more generalisable demographic data in terms of gestational ages, weights and respiratory support required by the infants. Another limitation is that the corrected gestational age at the time of the first KMC session was later than the aim of 28 weeks CGA originally set out in the research protocol. The median corrected gestational age at session one was 32^+6^ weeks. Another limitation that could impact our results is that there was often a delay between the first and second sessions of KMC. The median age for session two was 33^+2^ weeks. It is possible that physiological stability at a later corrected gestational age could have affected results and thus having the median age of both KMC sessions below 30 weeks’ gestation would have been beneficial data to capture. One reason for the delay in commencing KMC monitored sessions for this study was parental availability. As the study was designed to ensure monitoring only took place when a baby was having KMC as part of their routine care, this meant capturing a session depended on parental availability and on timing sessions with nursing and cares. We did not objectively capture parental experiences and preferences of KMC at 30° and 60° angles. As there was no statistically significant difference between the two angles, this could play a bigger role in the selection of KMC position.

## Conclusion

To the best of our knowledge our study represents the first to employ NIRS monitoring to examine if the angle of held position influences infants rSO_2_ during KMC. At present there is insufficient evidence to state whether one held position over another during kangaroo mother care is advantageous. However, whilst no difference was observed between either group, in our study we have found that neither position had a negative effect on infants. We propose further studies are required in this area to fully establish if there is an advantageous position in which to hold newborn infants during KMC.

## Supplementary information

Supplementary Figure 1

## Data Availability

Anonymised datasets are available upon written request from the corresponding author.
