# Supplementary material for: The effect of maternal position on cerebral oxygenation in premature infants during Kangaroo care: a randomised controlled trial
Source: J Perinatol. 2025 Apr 5;45(11):1552–7. doi: 10.1038/s41372-025-02287-0 (PMC12660145; doi:10.1038/s41372-025-02287-0)

## **Supplemental Data**

**Supplementary Figure 1:** Mauro Metal Relax chair with dimensions and incline illustration. The angle between the seat and the back rest ranges from 97° (seating position) to 120° (equating to the 60° incline used in the study) to an incline of just under 30° from the horizontal plane. (From Mauro Ergo-line technical file, accessed at:

<https://www.haelvoet.com/en/products/details/354>)


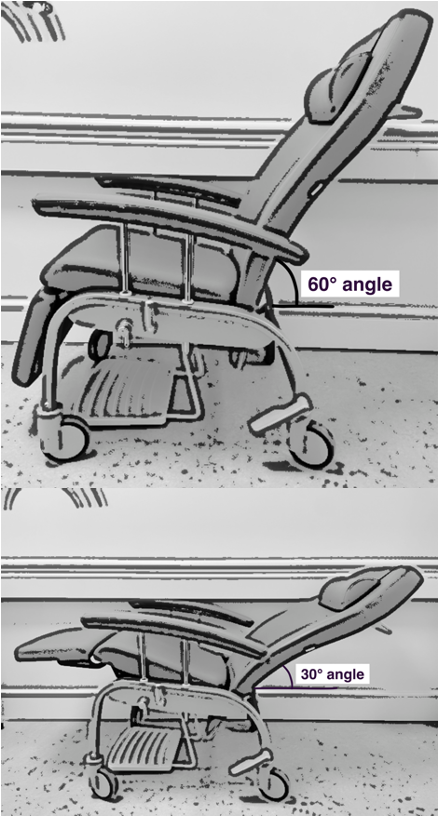

Supplement: Supplementary file 1 — Supplementary Figure 1 [file 41372_2025_2287_MOESM1_ESM.docx]
